# Review of studies mapping from quality of life or clinical measures to EQ-5D: an online database

**DOI:** 10.1186/1477-7525-11-151

**Published:** 2013-09-05

**Authors:** Helen Dakin

**Affiliations:** 1 Health Economics Research Centre, Nuffield Department of Population Health, University of Oxford, Old Road Campus, Headington, Oxford OX3 7LF, UK

**Keywords:** EuroQoL, EQ-5D, Utility, Mapping, Crosswalking, Health-related quality of life

## Abstract

Systematic literature searches were conducted to identify studies that conducted statistical mapping to predict EQ-5D utilities or responses from any source instrument and reported the estimated algorithms in sufficient detail to allow other researchers to use them to predict EQ-5D in other studies. Ninety studies reporting 121 mapping algorithms met the inclusion criteria. The studies estimated EQ-5D utilities from 80 source instruments. All but two studies included direct utility mapping to predict EQ-5D utilities, while 20 studies (22%) conducted response mapping to predict responses to each EQ-5D domain. Seventy-two studies (80%) explored ordinary least squares regression and 16 (18%) used censored least absolute deviations (CLAD) models. The details of the studies identified are made available in an online database, which will be updated regularly to enable researchers to easily identify studies that can help them to estimate utilities for economic evaluation.

## Introduction

Estimation of quality-adjusted life-years (QALYs) for economic evaluation requires data on health-related quality of life on a preference-based “utility” scale that captures preferences about the values of different health states on a scale from one (perfect health) through zero (death) to negative values (states worse than dead). Although several such preference-based quality of life measures have been developed, EQ-5D (EuroQoL) is the most commonly used [1] and some health technology assessment organisations (including the UK National Institute for Health and Care Excellence, NICE [2]) specifically request that EQ-5D is used in all economic evaluations submitted to them to ensure comparability between studies.

Since many studies collect data on non-preference-based measures of quality of life or clinical symptoms, but not EQ-5D, there is substantial demand for mapping algorithms that use statistical analyses to predict EQ-5D responses or utilities from responses or scores on other measures. Although other reviews of mapping studies have been conducted previously [3-8], at least 40 studies have been published since even the most recent study [7] was conducted and many older studies are also omitted from these previous reviews. An up-to-date, publicly-available database of mapping studies would help researchers to identify studies mapping between the instruments of interest, which is currently difficult, as there is no specific Medical Subject Headings (MeSH) term, several different terms used in the literature (e.g. mapping, mapped, crosswalk and regression) and many studies are published only as discussion or conference papers. It is also often difficult to identify studies extending the results of previous mapping studies by refining model specification, conducting external validation or developing tools to estimate predictions. This study therefore aimed to conduct a structured literature review identifying studies mapping to EQ-5D, which will be updated regularly and made publicly available.

## Review

### Methods

Systematic literature searches were conducted to identify all studies mapping to EQ-5D. Medline (via Pubmed), the Centre for Reviews and Dissemination (CRD, http://www.crd.york.ac.uk/crdweb) database (which includes DARE, NHS EED and HTA) and the Health Economists’ Study Group website (http://www.hesg.org.uk) were searched in December 2012 and in July 2013 using key words that included “EQ-5D” alongside “mapping”, “mapped”, “crosswalk” or “transfer to utility”. The EuroQoL Reference Search (http://www.euroqol.org) was searched in April and July 2013. The reference lists of previous systematic reviews of mapping studies, estimation of utilities or use of mapping in health technology assessment [3-8] were also reviewed. EMBASE was not searched due to resource constraints and as most studies that are in EMBASE but not Medline comprise conference abstracts that are less likely to report coefficients for mapping models. Studies meeting the following inclusion/exclusion criteria were included:

•Conduct statistical mapping (including, but not limited to, regression analyses) to predict EQ-5D utilities and/or responses from any source instrument. Studies using judgement mapping (in which researchers, experts or patients make judgements about how EQ-5D items relate to those on the source instrument) were excluded. Studies that simply reported the mean EQ-5D utility for different subgroups categorised by the source instrument were excluded unless such data were specifically reported with the intention of being used for mapping.

•Report algorithms or coefficients in sufficient detail that other researchers can use them to predict EQ-5D utilities and/or responses in other studies.

•No restrictions by publication date or status were imposed providing that the article was available in English.

•Papers validating an existing mapping algorithm or developing tools to estimate predictions were identified and linked to the source study in the online database but were not counted as separate studies unless they also estimated new mapping models not reported previously. Similarly, early versions of articles (e.g. conference or discussion papers) meeting inclusion criteria were not counted separately unless they presented additional mapping algorithms; instead such studies are linked to the final version in the online database.

•Valuation studies directly estimating health state valuations (e.g. using visual analogue, time trade-off or standard gamble) rather than EQ-5D were excluded.

Data were extracted on: citation details; the source instrument(s); the clinical area (classified using ICD-10 chapter headings); the number of paired observations of EQ-5D and source instruments used to estimate mapping models; and the types of statistical model/method evaluated. Where necessary, authors were contacted for clarification or additional information. All model types/specifications evaluated were recorded, regardless of which were considered to fit the data best or had coefficients reported. In the narrative review, key statistics are presented either by paper or by mapping algorithm, where a mapping algorithm is defined as prediction of EQ-5D from one source measure (or set of measures) to EQ-5D using a given dataset. For simplicity, mapping algorithms reported in the same paper that map from the same source instrument to the same target instrument are counted only once, regardless of how many different model specifications were explored or presented. A study mapping from SF-12 to EQ-5D and from SF-36 to EQ-5D using both ordinary least squares (OLS) and Tobit regression is therefore counted as two mapping algorithms (one from SF-12 to EQ-5D and one from SF-36). To simplify data extraction, no quality assessment was conducted.

### Results

**Figure 1:**
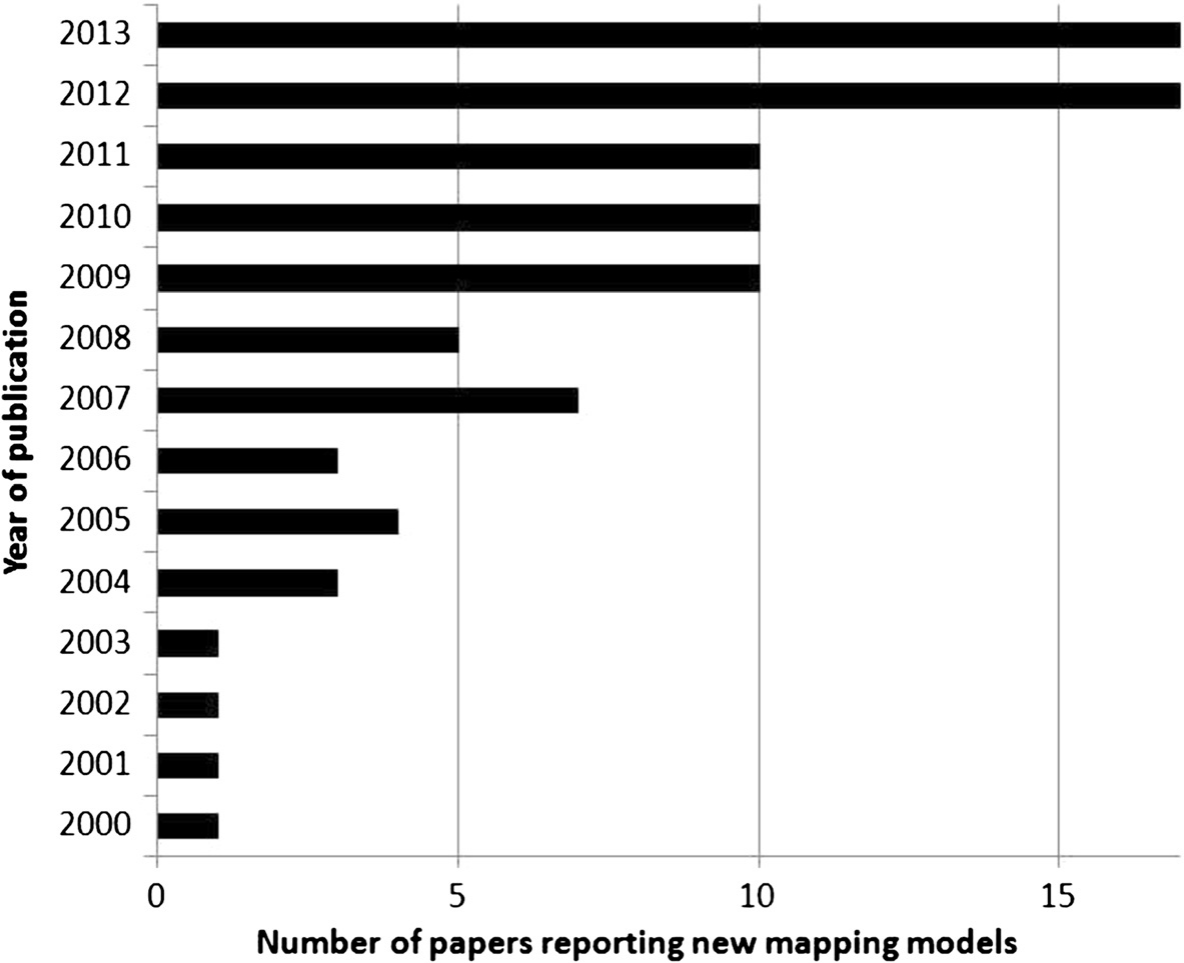
Number of mapping papers by year of publication.

Ninety studies reporting 121 mapping algorithms met inclusion criteria. Fourteen other identified studies were excluded as they did not report regression coefficients [9-22]. Full details of all included studies are available at: http://www.herc.ox.ac.uk/downloads/mappingdatabase. The number of studies published each year has increased substantially (Figure 1) over the past 15 years from one study per year in 2000–2003 to 17 studies in 2012 and 2013. The steepest increases occurred in 2009 and 2012, which follow the publication of the 2008 NICE methods guide endorsing mapping in the absence of directly measured EQ-5D [23] and the technical support document giving guidance on mapping methodology [24].

All but two [25,26] of the 90 included studies used some form of direct utility mapping, in which a statistical model predicts EQ-5D utility. Twenty studies (22%) [25,27-39] estimated response mapping models (also known as “indirect” mapping), in which EQ-5D responses for each of the five domains are predicted using categorical regression techniques (e.g. multinomial logistic regression) or cross-tabulation, thereby giving mapping models that can be used with any EQ-5D tariff.

Seventy-two studies (80%) evaluated linear models assuming Gaussian residuals (e.g. OLS), of which 35 (39%) studies evaluated only linear models, and 37 (41%) evaluated these alongside other specifications. Fourteen studies (16%) used Tobit models and 16 (18%) used censored least absolute deviations (CLAD) to allow for censoring. Generalised linear models (GLM) were used in eight studies (9%) and two-part models (such as those where one model predicted the probability of having perfect health and a second predicted utility conditional on having imperfect health) were used in nine studies (10%). Three studies did not clearly state their methods, while 40 used more than one model type.

Various other model specifications were also evaluated, including: latent class or mixture models [40-43]; spline or locally piecewise regression [43,44]; fractional polynomial regression [45,46]; generalised least squares [47,48]; fractional logit [27,33]; random-effects or mixed models [33,49]; and median regression [50]. Dakin et al. [27] developed a three-part model, which comprised a multinomial logit to predict the probability of having perfect health or having severe problems on any domain (N3), followed by two models predicting utility conditional on having moderate problems, or on having severe problems. Rowen et al. obtained visual analogue scale valuations and responses on different questionnaires and used OLS to map each instrument to visual analogue and then onto one another [51]. van Hout et al. also explored non-parametric cross-tabulation and a psychometric scaling approach, which uses item response theory to implement response mapping [38]. Richardson et al. [52] used geometric mean squares, which allows for the error in both the source and target instrument and ensures that models are logically coherent [19]. Among the studies excluded from the review due to not reporting sufficient information to estimate predicted EQ-5D utility, two used Bayesian probability networks [10,18]; this technique gave models with extremely good prediction accuracy, although it is unclear whether it is practical to publish such algorithms for use in other studies.

Eighty different source instruments (including combinations of two or more instruments or subscales) were mapped to EQ-5D. The Health Assessment Questionnaire (HAQ) was the most commonly mapped source instrument (15 models; Table 1). Thirteen models estimated EQ-5D utilities from SF-6D, SF-12 or SF-36 and a further 12 mapped from one of the European Organization for Research and Treatment of Cancer (EORTC) quality of life questionnaires. Eight source further instruments were each mapped in more than two studies (Table 1). In total, 33 models (27%) mapped from generic (rather than condition-specific) measures, in contrast to 63% of the studies identified by Brazier et al. [3] and 10% of recent NICE appraisals using mapping [6].

**Table 1:** Summary of the studies meeting inclusion criteria

	**Number (%) of mapping models**
Total	121
Source instrument*	
Barthel index	3 (2%)
28-joint disease activity score (DAS 28)	3 (2%)
Dermatology Life Quality Index (DLQI)	3 (2%)
European Organization for Research and Treatment of Cancer Quality of Life Questionnaire (EORTC QLQ), all variants	12 (10%)
Functional Assessment of Cancer Therapy (FACT), all variants	5 (4%)
HAQ (including modified HAQ and HAQ-DI)	15 (12%)
Modified Rankin scale (mRS)	3 (2%)
Numerical rating scale or visual analogue	4 (3%)
Parkinson’s Disease Questionnaire (PDQ-8 & PDQ-39)	5 (4%)
Short form variants (SF-6D, SF-12, SF-36)	13 (11%)
25-item Visual Functioning Questionnaire (VFQ-25)	3 (2%)
Other generic measures	15 (12%)
Other condition specific measures	41 (34%)
***Disease area***	
Cancer	16 (13%)
Cardiovascular	11 (9%)
Central nervous system	12 (10%)
Digestive system	5 (4%)
Eye conditions	3 (2%)
General population	16 (13%)
Mental health and behavioural disorders	4 (3%)
Musculoskeletal	28 (23%)
Respiratory system	3 (2%)
Skin	3 (2%)
Various	14 (12%)
Other	6 (5%)
***Sample size*** (number of observations in the estimation sample, including repeated measurements of the same patients)	
< 200	21 (17%)
200-499	16 (13%)
500-999	13 (11%)
1000-4999	27 (22%)
5000-19,999	19 (16%)
20,000-99,999	4 (3%)
≥ 100,000	2 (2%)
Not clearly stated	19 (16%)

*Numbers do not sum to the total as four studies predicted EQ-5D based on two instruments simultaneously (e.g. fitting a model predicting EQ-5D based on questions from both FACT and EORTC).

Sixteen mapping models (13%) were estimated on general population samples and 14 (12%) included mixed samples with a variety of conditions (Table 1). Twenty-eight studies (23%) were on musculoskeletal disease, which may, at least in part, be driven by the number of large datasets and NICE appraisals in this area. The remaining studies covered most ICD-10 chapters, although only two studies covered infectious disease and none examined haematological, obstetric or gynaecological conditions. *Value in Health* comprised the journal publishing the greatest number of mapping studies (20 studies) followed by *Quality of Life Research* (10 studies) and *Medical Decision Making* and *Health and Quality of Life Outcomes* (nine studies each). Many of the remaining studies were reported in disease-specific clinical journals.

The sample size used to estimate mapping models ranged from 48 to 134,269 observations, with median 1,167 and mean 6,069 (standard deviation: 17,289); 21 algorithms (17%) were estimated using < 200 observations (Table 1) and 16 (13%) used ≥10,000. For 19 mapping algorithms, the number of observations used to estimate mapping models was not clearly stated: generally as repeated observations of an unstated number of participants were included in the estimation sample.

### Conclusions

The database of mapping studies available at http://www.herc.ox.ac.uk/downloads/mappingdatabase (Additional file 1) provides researchers planning cost-utility analyses with an easy-to-access resource that helps them to identify mapping algorithms linking the instruments of interest. Details of any studies validating other mapping algorithms and any supplementary information downloadable from other sites, such as tools available to generate predictions, are also given. The database will be updated regularly as new studies are published and in the future may be extended to include studies mapping to other measures and/or extract data on additional fields if funding becomes available. The database may help with study identification in future reviews of utility estimation in specific disease areas and for reviewers and editors assessing the novelty of a new mapping algorithm. The database may also be useful for researchers developing a new mapping algorithm or validating an existing one to avoid duplicate publication and identify best practice. Duplication of previous work appears to be common, with 49% of mapping models using a source instrument that had been evaluated in a previous mapping study, although some duplication may be appropriate if the relationship between instruments differs between disease areas or if the new study uses superior methods or data. For HAQ, for example, two studies used mixture models [41,42], although the remainder used linear models with or without polynomial terms and/or random effects, and frequently generated very similar mapping models.

However, as with all studies, mapping models vary in quality and relevance to particular clinical problems. In particular, 17% of mapping models used < 200 observations and 39% of studies used OLS alone, which assumes that EQ-5D utilities are normally distributed with no ceiling effect, but often gives good prediction accuracy. Studies also varied substantially in prediction accuracy and in the completeness with which the estimation sample and model selection procedures were described. Researchers considering using a particular mapping algorithm in their study should review the quality of the study(ies) linking the instruments of interest (e.g. using [6]) and assess whether the sample(s) used in the development or validation of the algorithm in question encompasses the type of patients included in their study, to avoid extrapolating beyond the estimation sample [6]. At present, there is relatively little evidence on the extent to which mapping algorithms developed in one population generalise to another and general population samples may not provide sufficient observations with poor quality of life to accurately estimate utilities across the entire range. Sample size, availability of additional covariates and suitability of the algorithm to be used with secondary data may comprise additional considerations that may affect the choice of which algorithm to use in any given study.

By searching a wide range of databases up to early 2013, my review identified 90 studies, of which only 39 (43%) were identified in previous reviews. Most of the studies identified for the first time here were either published in 2011–2013 or were economic evaluations that also developed mapping algorithms. The main limitation of this review is that EMBASE was not searched; as result, some studies (e.g. conference abstracts) may have been missed. Furthermore, some studies reported only in the grey literature (such as reports for funders, NICE manufacturer submissions or conference papers) may have been missed. Coefficients may also be available from the authors for some studies that were excluded from the review on the grounds that the algorithms were not published in sufficient detail to allow them to be used in other studies.

The review also suggests that the more complex model specifications (e.g. response mapping or mixture models) are gaining in popularity and highlights a variety of model specifications that have been used in a small number of studies and warrant further investigation. Mixture models [41], response mapping and non-parametric cross-tabulation [38] may be particularly useful for allowing for the non-Gaussian distribution of utilities when the sample size is sufficiently large. Improvements to study reporting are also warranted: in particular, researchers should clearly report the number of observations included in the estimation sample and ensure that the full mapping algorithm is made available for other researchers to use. Finally, mapping should always be considered second best to direct EQ-5D measurement, since it introduces additional errors and assumptions: the existence of published mapping algorithms is no substitute for inclusion of appropriate utility instruments within clinical trials.

## Abbreviations

CLAD: Censored least absolute deviations; CRD: Centre for Reviews and Dissemination; EORTC: European Organization for Research and Treatment of Cancer; GLM: Generalised linear models; HAQ: Health Assessment Questionnaire; MeSH: Medical Subject Headings; NICE: National Institute for Health and Care Excellence; OLS: Ordinary least squares; QALY: Quality-adjusted life-year.

## Competing interests

The author declares that she has no competing interest.

## Supplementary Material

Additional file 1 **The master version of the database is available at**http://www.herc.ox.ac.uk/downloads/mappingdatabase**and will be updated regularly as more studies are published.** Click here for file
