# Supplementary material for: Review of studies mapping from quality of life or clinical measures to EQ-5D: an online database
Source: Health Qual Life Outcomes. 2013 Sep 5;11:151. doi: 10.1186/1477-7525-11-151 (PMC3844400; doi:10.1186/1477-7525-11-151)
Supplement: Additional file 1 — The master version of the database is available athttp://www.herc.ox.ac.uk/downloads/mappingdatabaseand will be updated regularly as more studies are published. [file 1477-7525-11-151-S1.doc]

**Full data extraction table**

The master version of the database is available at http://www.herc.ox.ac.uk/downloads/mappingdatabase and will be updated regularly as more studies are published.

| **Citation details** | **Quality of life measures** | | **Disease or patient group** | **Disease category** | **No. observations in estimation sample** | **Mapping models investigated** |
| --- | --- | --- | --- | --- | --- | --- |
|  | **From** | **To** |  |  |  |  |
| Adams R., Walsh C., Veale D., Bresnihan B., FitzGerald O., Barry M. (2010). Understanding the relationship between the EQ-5D, SF-6D, HAQ and disease activity in inflammatory arthritis. Pharmacoeconomics. 28 (6), 477-87. | Health Assessment Questionnaire (HAQ) | EQ-5D | Rheumatoid arthritis | Musculoskeletal | 345 pts | OLS |
|  |  | EQ-5D | Psoriatic arthritis | Musculoskeletal | 159 pts | OLS |
|  | 28-joint disease activity score (DAS 28) developed by European League Against Arthritis (EULAR) | EQ-5D | Rheumatoid arthritis | Musculoskeletal | 345 pts | OLS |
|  |  | EQ-5D | Psoriatic arthritis | Musculoskeletal | 159 pts | OLS |
|  | Health Assessment Questionnaire (HAQ) | SF-6D | Rheumatoid arthritis | Musculoskeletal | 345 pts | OLS |
|  |  | SF-6D | Psoriatic arthritis | Musculoskeletal | 159 pts | OLS |
|  | 28-joint disease activity score (DAS 28) developed by European League Against Arthritis (EULAR) | SF-6D | Rheumatoid arthritis | Musculoskeletal | 345 pts | OLS |
|  |  | SF-6D | Psoriatic arthritis | Musculoskeletal | 159 pts | OLS |
| Adams R., Craig B. M., Walsh C. D., Veale D. J., Bresnihan B., FitzGerald O., et al. (2011). The impact of a revised EQ-5D population scoring on preference-based utility scores in an inflammatory arthritis cohort. Value Health. 14 (6), 921-7. | Health Assessment Questionnaire (HAQ) | EQ-5D | Rheumatoid arthritis | Musculoskeletal | 345 pts | OLS |
|  |  | EQ-5D | Psoriatic arthritis | Musculoskeletal | 159 pts | OLS |
|  | 28-joint disease activity score (DAS 28) developed by European League Against Arthritis (EULAR) | EQ-5D | Rheumatoid arthritis | Musculoskeletal | 345 pts | OLS |
| Ara R. M., Reynolds A. V., Conway P. (2007). The cost-effectiveness of etanercept in patients with severe ankylosing spondylitis in the UK. Rheumatology (Oxford). 46 (8), 1338-44. | Bath Ankylosing Spondylitis Disease Activity Index (BASDAI) and Bath Ankylosing Spondylitis Functional Index (BASFI) | EQ-5D | Ankylosing Spondylitis | Musculoskeletal | Unclear | Not stated |
| Ara R., Brazier J. (2008). Deriving an algorithm to convert the eight mean SF-36 dimension scores into a mean EQ-5D preference-based score from published studies (where patient level data are not available). Value Health. 11 (7), 1131-43. | SF-36 | EQ-5D | Asthma, chest pain, older people, COPD, irritable bowel syndrome, trauma, back pain, leg disorders, osteoarthritis | Various | 6,350 | OLS |
| Askew R. L., Swartz R. J., Xing Y., Cantor S. B., Ross M. I., Gershenwald J. E., et al. (2011). Mapping FACT-melanoma quality-of-life scores to EQ-5D health utility weights. Value Health. 14 (6), 900-6. | Functional assessment of Cancer Therapy-Melanoma (FACT-M) | EQ-5D | Melanoma | Cancer | 138 | OLS; CLAD |
| Badia, X., Roset, M., Valassi, E., Franz, H., Forsythe, A., & Webb, S. M. (2013). Mapping CushingQOL scores to EQ-5D utility values using data from the European Registry on Cushing's syndrome (ERCUSYN). Qual Life Res. | CushingQOL | EQ-5D | Cushing’s syndrome | Endocrine disorders | 128 | GLM; Tobit |
| Bansback N, Marra C, Tsuchiya A, Anis A, Guh D, Hammond T, et al. Using the health assessment questionnaire to estimate preference-based single indices in patients with rheumatoid arthritis. Arthritis Rheum. 2007 Aug 15;57(6):963-71. | Health Assessment Questionnaire Disability Index (HAQ-DI) | EQ-5D | Rheumatoid arthritis | Musculoskeletal | 439 | GEE |
|  |  | SF-6D | Rheumatoid arthritis | Musculoskeletal | 439 | GEE |
| Barton G. R., Sach T. H., Jenkinson C., Avery A. J., Doherty M., Muir K. R. (2008). Do estimates of cost-utility based on the EQ-5D differ from those based on the mapping of utility scores? Health Qual Life Outcomes. 6, 51. | Western Ontario and McMaster Universities Osteoarthritis Index (WOMAC) | EQ-5D | Knee pain | Musculoskeletal | 259 | OLS |
| Blome, C., Beikert, F. C., Rustenbach, S. J., & Augustin, M. (2013). Mapping DLQI on EQ-5D in psoriasis: transformation of skin-specific health-related quality of life into utilities. Arch Dermatol Res, 305(4), 197-204. | Dermatology Life Quality Index (DLQI) and clinical indicators | EQ-5D | Psoriasis | Skin | 1511 | OLS |
| Brennan D. S., Spencer A. J. (2006). Mapping oral health related quality of life to generic health state values. BMC Health Serv Res. 6, 96. | Oral Health Impact Profile (OHIP) | EQ-5D | Dental patients | General population | 250 | Tobit |
| Browne C., Brazier J., Carlton J., Alavi Y., Jofre-Bonet M. (2012). Estimating quality-adjusted life years from patient-reported visual functioning. Eye (Lond). 26 (10), 1295-301. | 25-item Visual Functioning Questionnaire (VFQ-25) | EQ-5D | Glaucoma | Eye conditions | 131 patients | OLS; CLAD; Tobit |
|  |  | SF-6D | Glaucoma | Eye conditions | 124 patients | OLS; CLAD; Tobit |
| Buxton M. J., Lacey L. A., Feagan B. G., Niecko T., Miller D. W., Townsend R. J. (2007). Mapping from disease-specific measures to utility: an analysis of the relationships between the Inflammatory Bowel Disease Questionnaire and Crohn's Disease Activity Index in Crohn's disease and measures of utility. Value Health. 10 (3), 214-20. | Inflammatory Bowel Disease Questionnaire (IBDQ) | EQ-5D | Crohn's disease | Digestive system | 3,320 | Linear mixed models |
|  | Crohn's Disease Activity Index (CDAI) | EQ-5D | Crohn's disease | Digestive system | 3575 | Linear mixed models |
|  | Inflammatory Bowel Disease Questionnaire (IBDQ) | SF-6D | Crohn's disease | Digestive system | 3,230 | Linear mixed models |
|  | Crohn's Disease Activity Index (CDAI) | SF-6D | Crohn's disease | Digestive system | 3640 | Linear mixed models |
| Calvert M. J., Freemantle N., Yao G., Cleland J. G., Billingham L., Daubert J. C., et al. (2005). Cost-effectiveness of cardiac resynchronization therapy: results from the CARE-HF trial. European heart journal. 26 (24), 2681-8. | Minnesota Living with Heart Failure Questionnaire (MLWHF) | EQ-5D | Heart failure | Cardiovascular | 813 patients | Linear mixed models |
| Carreno A., Fernandez I., Badia X., Varela C., Roset M. (2011). Using HAQ-DI to estimate HUI-3 and EQ-5D utility values for patients with rheumatoid arthritis in Spain. Value Health. 14 (1), 192-200. | Health Assessment Questionnaire Disability Index (HAQ-DI) | EQ-5D | Rheumatoid arthritis | Musculoskeletal | 235 | OLS |
|  |  | HUI3 | Rheumatoid arthritis | Musculoskeletal | 206 | OLS |
| Cheung Y. B., Tan L. C., Lau P. N., Au W. L., Luo N. (2008). Mapping the eight-item Parkinson's Disease Questionnaire (PDQ-8) to the EQ-5D utility index. Qual Life Res. 17 (9), 1173-81. | 8-item Parkinson’s Disease Questionnaire (PDQ-8) | EQ-5D | Parkinson's disease | Central nervous system | 162 | OLS; CLAD |
| Cheung Y. B., Thumboo J., Gao F., Ng G. Y., Pang G., Koo W. H., et al. (2009). Mapping the English and Chinese versions of the Functional Assessment of Cancer Therapy-General to the EQ-5D utility index. Value Health. 12 (2), 371-6. | Functional Assessment of Cancer Therapy-General (FACT-G) | EQ-5D | Cancer | Cancer | 572 | OLS; CLAD |
| Crott R., Briggs A. (2010). Mapping the QLQ-C30 quality of life cancer questionnaire to EQ-5D patient preferences. Eur J Health Econ. 11 (4), 427-34. | EORTC Quality of Life Questionnaire (QLQ-C30) | EQ-5D | Breast cancer | Cancer | Around 800 | OLS |
| Currie C. J., Conway P. (2007). Evaluation of the association between EQ5D utility and dermatology life quality index (DLQI) score in patients with psoriasis. Value Health. 10 (6), A470-1 (Abstract PSK11). | Dermatology Life Quality Index (DLQI) | EQ-5D | Psoriasis | Skin | 94 | Not stated |
| Dakin, H., Gray, A., & Murray, D. (2013). Mapping analyses to estimate EQ-5D utilities and responses based on Oxford Knee Score. Qual Life Res, 22(3), 683-694. | Oxford Knee Score (OKS) | EQ-5D | Knee replacement for arthritis | Musculoskeletal | 134,269 | OLS; GLM; 2-part; response mapping; 3-part |
| Dams, J., Klotsche, J., Bornschein, B., Reese, J. P., Balzer-Geldsetzer, M., Winter, Y., Schrag, A., Siderowf, A., Oertel, W. H., Deuschl, G., Siebert, U., & Dodel, R. (2013). Mapping the EQ-5D index by UPDRS and PDQ-8 in patients with Parkinson's disease. Health Qual Life Outcomes, 11, 35. | Unified Parkinson’s Disease Rating Scale (UPDRS) | EQ-5D | Parkinson’s disease | central nervous system | 121 | OLS; fractional polynomial regression; logarithmic function |
|  | 8-item Parkinson’s Disease Questionnaire (PDQ-8) | EQ-5D | Parkinson’s disease | central nervous system | 121 |  |
| Eldin R., Tsuchiya A., Brazier J. (2002). Mapping the Minnesota Living with Heart Failure Questionnaire to the EQ-5D index. Report to Novartis Contact authors for further details. | Minnesota Living with Heart Failure Questionnaire (MLWHF) | EQ-5D | NYHA Class II-IV heart failure patients | Cardiovascular | 22,931 | OLS; response mapping |
| Franks P., Lubetkin E. I., Gold M. R., Tancredi D. J. (2003). Mapping the SF-12 to preference-based instruments: convergent validity in a low-income, minority population. Med Care. 41 (11), 1277-83. | SF-12 | EQ-5D | Low income ethnic minorities | General population | 240 | OLS |
|  |  | HUI3 | Low income ethnic minorities | General population | 240 | OLS |
| Franks P., Lubetkin E. I., Gold M. R., Tancredi D. J., Jia H. (2004). Mapping the SF-12 to the EuroQol EQ-5D Index in a national US sample. Med Decis Making. 24 (3), 247-54. | SF-12 | EQ-5D | General population | General population | 12,998 | OLS |
| Ghatnekar, O., Eriksson, M., & Glader, E. L. (2013). Mapping health outcome measures from a stroke registry to EQ-5D weights. Health Qual Life Outcomes, 11, 34. | Stroke outcome measures not restricted to validated instruments | EQ-5D | Stroke | Cardiovascular | 272 | OLS; CLAD; Tobit |
| Gillard P. J., Devine B., Varon S. F., Liu L., Sullivan S. D. (2012). Mapping from disease-specific measures to health-state utility values in individuals with migraine. Value Health. 15 (3), 485-94. | Headache Impact Test (HIT-6) | EQ-5D | Migraine | Central nervous system | 6,334 | OLS |
|  | Migraine-Specific Quality-of-Life Questionnaire version 2.1 (MSQ) | EQ-5D | Migraine | Central nervous system | 6,108 | OLS |
| Goldsmith K. A., Dyer M. T., Buxton M. J., Sharples L. D. (2010). Mapping of the EQ-5D index from clinical outcome measures and demographic variables in patients with coronary heart disease. Health Qual Life Outcomes. 8 (1), 54. | Clinical outcome measures and demographic variables, including Seattle Angina Questionnaire | EQ-5D | Cardiovascular disease | Cardiovascular | 2,855 | OLS |
| Gordon J., Lister S., Prettyjohns M., McEwan P., Tetlow A., Gabriel Z. (2012). A cost-utility study of the use of pregabalin in treatment-refractory neuropathic pain. J Med Econ. 15 (2), 207-18. | Numerical rating scale (NRS) of pain severity | EQ-5D | Neuropathic pain | Musculoskeletal | 284 | GLM |
| Gray A. M., Rivero-Arias O., Clarke P. M. (2006). Estimating the association between SF-12 responses and EQ-5D utility values by response mapping. Med Decis Making. 26 (1), 18-29. | SF-12 | EQ-5D | General population | General population | 12,967 | OLS; response mapping |
| Gu N. Y., Botteman M. F., Ji X., Bell C. F., Carter J. A., van Hout B. (2011). Mapping of the Insomnia Severity Index and other sleep measures to EuroQol EQ-5D health state utilities. Health Qual Life Outcomes. 9, 119. | Insomnia Severity Index (ISI) | EQ-5D | Adults with sleep problems | Mental health and behavioural disorders | 1421 | GLM |
| Gu N. Y., Bell C., Botteman M. F., Ji X., Carter J. A., van Hout B. (2012). Estimating preference-based EQ-5D health state utilities or item responses from neuropathic pain scores. Patient. 5 (3), 185-97. | 11-point pain intensity numerical rating scale (PI-NRS-11) | EQ-5D | Painful diabetic peripheral neuropathy or post-herpetic neuralgia | Musculoskeletal | 1903 | OLS; response mapping |
| Hawton A., Green C., Telford C. J., Wright D. E., Zajicek J. P. (2011). The use of multiple sclerosis condition-specific measures to inform health policy decision-making: mapping from the MSWS-12 to the EQ-5D. Mult Scler. 18 (6), 853-61. | 12-item Multiple Sclerosis Walking Scale (MSWS-12) | EQ-5D | Multiple sclerosis | Central nervous system | 560 | OLS; 2-part; CLAD; Tobit; 3-part |
| Hawton A., Green C., Telford C., Zajicek J., Wright D. (2012). Using the Multiple Sclerosis Impact Scale to Estimate Health State Utility Values: Mapping from the MSIS-29, Version 2, to the EQ-5D and the SF-6D. Value Health. 15 (8), 1084-91. | 29-item Multiple Sclerosis Impact Scale (MSIS-29) | EQ-5D | Multiple sclerosis | Central nervous system | 672 | OLS; CLAD; Tobit |
|  |  | SF-6D | Multiple sclerosis | Central nervous system | 495 | OLS; CLAD; Tobit |
| Hernández Alava M., Wailoo A. J., Ara R. (2012). Tails from the peak district: adjusted limited dependent variable mixture models of EQ-5D questionnaire health state utility values. Value Health. 15 (3), 550-61. | Health Assessment Questionnaire Disability Index (HAQ-DI) | EQ-5D | Rheumatoid arthritis | Musculoskeletal | 467 patients | OLS; Tobit; mixture model |
| Hernández Alava, M., Wailoo, A., Wolfe, F., & Michaud, K. (2013). The relationship between EQ-5D, HAQ and pain in patients with rheumatoid arthritis. Rheumatology (Oxford), 52, 944-950 and Hernández Alava, M., Wailoo, A., Wolfe, F., & Michaud, K. (2012). A comparison of direct and indirect methods for the estimation of health utilities from clinical outcomes. University of Sheffield, HEDS Discussion Paper Retrieved 3rd December 2012, from http://www.nicedsu.org.uk/Mapping%20of%20EQ-5D.DP.pdf | Health Assessment Questionnaire (HAQ) and pain on VAS | EQ-5D | Rheumatoid arthritis | Musculoskeletal | 100,398 | OLS; response mapping; limited dependent variable mixture model. Response mapping conducted using generalised ordered probit |
| Huang I. C., Frangakis C., Atkinson M. J., Willke R. J., Leite W. L., Vogel W. B., et al. (2008). Addressing ceiling effects in health status measures: a comparison of techniques applied to measures for people with HIV disease. Health Serv Res. 43 (1 Pt 1), 327-39. | Medical Outcomes Study Health-Related Quality of Life Measures in HIV/AIDS (MOS-HIV) | EQ-5D | HIV | Infectious disease | 1,013 | OLS; 2-part; CLAD; latent class model |
| Jang R. W., Isogai P. K., Mittmann N., Bradbury P. A., Shepherd F. A., Feld R., et al. (2010). Derivation of utility values from European Organization for Research and Treatment of Cancer Quality of Life-Core 30 questionnaire values in lung cancer. Journal of Thoracic Oncology. 5 (12), 1953-7. | EORTC Quality of Life Questionnaire (QLQ-C30) | EQ-5D | Lung cancer | Cancer | 172 | OLS |
| Jia H., Zack M. M., Moriarty D. G., Fryback D. G. (2011). Predicting the EuroQol Group's EQ-5D Index from CDC's "Healthy Days" in a US Sample. Med Decis Making. 31 (1), 174. | Healthy Days, developed by Centers for Disease Control and Prevention (CDC) | EQ-5D | General population | General population | 3844 | Spline regressions (locally piecewise polynomial regression) |
| Kaambwa, B., Billingham, L., & Bryan, S. (2013). Mapping utility scores from the Barthel index. Eur J Health Econ, 14(2), 231-241. | Barthel index | EQ-5D | Older people | General population | 793 | OLS; CLAD; response mapping |
| Kay, S., Tolley, K., Colayco, D., Khalaf, K., Anderson, P., & Globe, D. (2013). Mapping EQ-5D utility scores from the Incontinence Quality of Life Questionnaire among patients with neurogenic and idiopathic overactive bladder. Value Health, 16(2), 394-402. | Incontinence-specific Quality of Life questionnaire (I-QOL) | EQ-5D | Overactive bladder | Urogenital | 2605 | 2-part |
| Kent, S., McIntosh, E., Gray, A., Jenkinson, C., Clarke, C., Gray, R., Williams, A., & Wheatley, K. (2012). Mapping algorithms from the Parkinson’s Disease Questionnaire to EUROQoL EQ-5D. Poster presentation at the 14th Biennial European Meeting of the Society for Medical Decision Making, Oslo, Norway, June 10-12. Poster and regression coefficients are available from the authors on request. | 39-item Parkinson’s Disease Questionnaire (PDQ-39) | EQ-5D | Parkinson's disease | Central nervous system | 9123 | OLS; response mapping |
|  | 8-item Parkinson’s Disease Questionnaire (PDQ-8) | EQ-5D | Parkinson's disease | Central nervous system | 9123 |  |
| Kim E. J., Ko S. K., Kang H. Y. (2012). Mapping the cancer-specific EORTC QLQ-C30 and EORTC QLQ-BR23 to the generic EQ-5D in metastatic breast cancer patients. Qual Life Res. 21 (7), 1193-203. | EORTC Quality of Life Questionnaire (QLQ-C30) | EQ-5D | Breast cancer | Cancer | 149 | OLS |
|  | EORTC breast cancer instrument (QLQ-BR23) | EQ-5D | Breast cancer | Cancer | 149 | OLS |
|  | EORTC QLQ-C30 and EORTC QLQ-BR23 | EQ-5D | Breast cancer | Cancer | 149 | OLS |
|  | EORTC Quality of Life Questionnaire (QLQ-C30) | EQ-5D | Breast cancer | Cancer | 149 | OLS |
| Kim, S. H., Jo, M. W., Kim, H. J., & Ahn, J. H. (2012). Mapping EORTC QLQ-C30 onto EQ-5D for the assessment of cancer patients. Health Qual Life Outcomes, 10, 151. | EORTC Quality of Life Questionnaire (QLQ-C30) | EQ-5D | Cancer (28 different types, including breast and colorectal) | Cancer | 893 | OLS |
| Koltowska-Haggstrom M., Jonsson B., Isacson D., Bingefors K. (2007). Using EQ-5D to derive general population-based utilities for the quality of life assessment of growth hormone deficiency in adults (QoL-AGHDA). Value Health. 10 (1), 73-81. | QoL Assessment of Growth Hormone Deficiency in Adults questionnaire (QoLAGHDA) | EQ-5D | General population | General population | 1,714 | OLS |
| Kontodimopoulos N., Aletras V. H., Paliouras D., Niakas D. (2009). Mapping the cancer-specific EORTC QLQ-C30 to the preference-based EQ-5D, SF-6D, and 15D instruments. Value Health. 12 (8), 1151-7. | EORTC Quality of Life Questionnaire (QLQ-C30) | EQ-5D | Gastric cancer | Cancer | 48 | OLS |
|  |  | 15D | Gastric cancer | Cancer | 48 | OLS |
|  |  | SF-6D | Gastric cancer | Cancer | 48 | OLS |
| Kontodimopoulos, N., Bozios, P., Yfantopoulos, J., & Niakas, D. (2013). Longitudinal predictive ability of mapping models: examining post-intervention EQ-5D utilities derived from baseline MHAQ data in rheumatoid arthritis patients. Eur J Health Econ, 14(2), 307-14. | Modified Health Assessment Questionnaire (MHAQ) | EQ-5D | Rheumatoid arthritis | Musculoskeletal | 143 | OLS |
| Lawrence W. F., Fleishman J. A. (2004). Predicting EuroQoL EQ-5D preference scores from the SF-12 Health Survey in a nationally representative sample. Med Decis Making. 24 (2), 160-9. | SF-12 | EQ-5D | General population | General population | 7,313 | OLS |
| Lloyd, A., Nafees, B., Gavriel, S., Rousculp, M. D., Boye, K. S., & Ahmad, A. (2008). Health utility values associated with diabetic retinopathy. Diabet Med, 25(5), 618-624. | 25-item Visual Functioning Questionnaire (VFQ-25) and visual acuity | EQ-5D | Diabetes and diabetic retinopathy | Eye conditions | 148 | Not stated |
| Longo M., Cohen D., Hood K., Robling M. (2000). Deriving an 'enhanced' EuroQoL from SF-36. Presented at the Health Economics Study Group (HESG) meeting, July 2000, Nottingham. | SF-36 | EQ-5D | Breast disease | Various | 468 | OLS |
| Longworth L., Buxton M. J., Sculpher M., Smith D. H. (2005). Estimating utility data from clinical indicators for patients with stable angina. Eur J Health Econ. 6 (4), 347-53; Longworth, L. (May 2007). Estimating quality adjusted life years where health-related utility data are missing. PhD thesis. Brunel University. | Breathlessness Grade and Canadian Cardiovascular Society (CCS) classification of angina and number of drug classes used | EQ-5D | Coronary artery disease | Cardiovascular | 503 | OLS; Tobit; response mapping |
| Longworth, L. (2007). Estimating quality adjusted life years where health-related utility data are missing. PhD thesis. Brunel University. | SF-36 | EQ-5D | Coronary artery disease | Cardiovascular | 423 | Response mapping |
| Longworth, L., Yang, Y., Young, T., Mulhern, B., Hernández Alava, M., Mukuria, C., Rowen, D., Tosh, J., Tsuchiya, A., Evans, P., Keetharuth, A. D., & Brazier, J. (2013). Use of generic and condition-specific measures of health related quality of life in NICE decision-making. Health Technol Assess (submitted). Coefficients available from the authors on request. | EORTC QLQ-C30 | EQ-5D | Multiple myeloma, breast cancer, lung cancer | Cancer | 771 | OLS; 2-part; Tobit; response mapping; polynomial spline |
|  | Functional Assessment of Cancer Therapy-General (FACT-G) | EQ-5D | Cancer (various) | Cancer | 530 | OLS; 2-part; Tobit; response mapping; polynomial spline, limited dependent variable mixture model |
| Madan J., Khan K., Lamb S. E., Petrou S. (2013). A comparison of Bayesian and frequentist approaches for mapping between the Roland Morris Disability questionnaire and generic preference-based measures. Presented at the Health Economics Study Group (HESG) meeting, January 2013, Exeter, UK. | Roland Morris Disability Questionnaire (RMQ) | EQ-5D | Back pain | Musculoskeletal | 2188 | OLS; CLAD; response mapping; mixed models; fractional logit. OLS, fractional logit and response mapping were replicated using Bayesian MCMC. Transition model of change from baseline |
|  | Roland Morris Disability Questionnaire (RMQ) | SF-6D | Back pain | Musculoskeletal | 2138 | OLS; GLM; CLAD; response mapping; mixed models |
| Maund E., Craig D., Suekarran S., Neilson A., Wright K., Brealey S., et al. (2012). Management of frozen shoulder: a systematic review and cost-effectiveness analysis. Health Technol Assess. 16 (11), 1-264. | Visual analogue scale rating of pain | EQ-5D | Frozen shoulder | Musculoskeletal | 141 | OLS; CLAD; Tobit |
|  | SF-36 | EQ-5D | Frozen shoulder | Musculoskeletal | 133 | OLS; CLAD; Tobit |
| McDaid C., Griffin S., Weatherly H., Duree K., van der Burgt M., van Hout S., et al. (2009). Continuous positive airway pressure devices for the treatment of obstructive sleep apnoea-hypopnoea syndrome: a systematic review and economic analysis. Health Technol Assess. 13 (4), iii-iv, xi-xiv, 1-119, 43-274. | Epworth Sleepiness Scale (ESS) | EQ-5D | Sleep apnoea | Ear, nose and throat | Not stated | OLS; GLM |
|  |  | SF-6D | Sleep apnoea | Ear, nose and throat | Not stated | OLS; GLM |
| McKenzie L., van der Pol M. (2009). Mapping the EORTC QLQ C-30 onto the EQ-5D instrument: the potential to estimate QALYs without generic preference data. Value Health. 12 (1), 167-71. | EORTC Quality of Life Questionnaire (QLQ-C30) | EQ-5D | Oesophageal cancer | Cancer | 877 | OLS; response mapping |
| Michaud, K., & Wolfe, F. (2005). EQ5D changes rheumatoid arthritis quality of life in United States: A retrospective study of 11,289 patients. Arthritis Rheum, 52(Suppl), S400. | Health assessment questionnaire (HAQ) | EQ-5D | Rheumatoid arthritis | Musculoskeletal | 35,422 | OLS |
| Norlin, J. M., Steen Carlsson, K., Persson, U., & Schmitt-Egenolf, M. (2012). Analysis of three outcome measures in moderate to severe psoriasis: a registry-based study of 2450 patients. Br J Dermatol, 166(4), 797-802. | Dermatology Life Quality Index (DLQI) | EQ-5D | Psoriasis | Skin | 2,450 | OLS |
| Oppe M., Devlin N., Black N. (2011). Comparison of the underlying constructs of the EQ-5D and Oxford Hip Score: implications for mapping. Value Health. 14 (6), 884-91. | Oxford Hip Score (OHS) | EQ-5D | Hip replacement | Musculoskeletal | 919 | OLS |
| Parker M., Haycox A., Graves J. (2011). Estimating the relationship between preference-based generic utility instruments and disease-specific quality-of-life measures in severe chronic constipation: challenges in practice. Pharmacoeconomics. 29 (8), 719-30. | Patient Assessment of Constipation quality of life (PAC-QOL) and symptom (PAC-SYM) scores | EQ-5D | Constipation | Digestive system | 5488 | GLS |
|  |  | SF-6D | Constipation | Digestive system | 5488 | GLS |
| Payakachat N., Summers K. H., Pleil A. M., Murawski M. M., Thomas J., 3rd, Jennings K., et al. (2009). Predicting EQ-5D utility scores from the 25-item National Eye Institute Vision Function Questionnaire (NEI-VFQ 25) in patients with age-related macular degeneration. Qual Life Res. 18 (7), 801-13. | 25-item National Eye Institute Vision Function Questionnaire (NEI-VFQ 25) | EQ-5D | Age-related macular degeneration | Eye conditions | 151 | OLS; CLAD; Tobit |
| Pinedo-Villanueva, R. A., Turner, D., Judge, A., Raftery, J. P., & Arden, N. K. (2013). Mapping the Oxford hip score onto the EQ-5D utility index. Qual Life Res, 22(3), 665-675. | Oxford Hip Score (OHS) | EQ-5D | Hip replacement | Musculoskeletal | 3518 | OLS; 2-part; Response mapping |
| Poole C. D., Connolly M. P., Nielsen S. K., Currie C. J., Marteau P. (2010). A comparison of physician-rated disease severity and patient reported outcomes in mild to moderately active ulcerative colitis. J Crohns Colitis. 4 (3), 275-82. | Physician-rated ulcerative colitis disease activity index (UCDAI) | EQ-5D | Ulcerative colitis | Digestive system | 326 | Response mapping |
| Revicki D. A., Kawata A. K., Harnam N., Chen W. H., Hays R. D., Cella D. (2009). Predicting EuroQol (EQ-5D) scores from the patient-reported outcomes measurement information system (PROMIS) global items and domain item banks in a United States sample. Qual Life Res. 18 (6), 783-91. | Patient-reported outcomes measurement information system (PROMIS) | EQ-5D | General population and various diseases | Various | 6,975 | OLS |
| Richardson, J., Iezzi, A., Khan, M., & Maxwell, A. (2012). Cross-national comparison of twelve quality of life instruments, MIC Papers 2-7. Monash Centre for Health Economics Research Papers 78, 80-83 and 85. Available at: http://www.buseco.monash.edu.au/centres/che/che-publications.html | HUI3 | EQ-5D | General population and asthma, cancer, depression, diabetes, hearing problems, arthritis, heart disease, COPD and stroke | Various | 1177-1467 | Geometric mean squares (GMS) |
|  | SF-6D | EQ-5D |  | Various | 1177-1467 |  |
|  | 15D | EQ-5D |  | Various | 1177-1467 |  |
|  | QWB | EQ-5D |  | Various | 1177-1467 |  |
|  | AQoL-4D | EQ-5D |  | Various | 1177-1467 |  |
|  | AQoL-8D | EQ-5D |  | Various | 1177-1467 |  |
|  | Personal Wellbeing Index (PWI) | EQ-5D |  | Various | 1177-1467 |  |
|  | Satisfaction with Life Survey (SWLS) | EQ-5D |  | Various | 1177-1467 |  |
|  | EQ-5D | HUI3 |  | Various | 1177-1467 |  |
|  | SF-6D | HUI3 |  | Various | 1177-1467 |  |
|  | 15D | HUI3 |  | Various | 1177-1467 |  |
|  | QWB | HUI3 |  | Various | 1177-1467 |  |
|  | AQoL-4D | HUI3 |  | Various | 1177-1467 |  |
|  | AQoL-8D | HUI3 |  | Various | 1177-1467 |  |
|  | Personal Wellbeing Index (PWI) | HUI3 |  | Various | 1177-1467 |  |
|  | Satisfaction with Life Survey (SWLS) | HUI3 |  | Various | 1177-1467 |  |
|  | EQ-5D | SF-6D |  | Various | 1177-1467 |  |
|  | HUI3 | SF-6D |  | Various | 1177-1467 |  |
|  | 15D | SF-6D |  | Various | 1177-1467 |  |
|  | QWB | SF-6D |  | Various | 1177-1467 |  |
|  | AQoL-4D | SF-6D |  | Various | 1177-1467 |  |
|  | AQoL-8D | SF-6D |  | Various | 1177-1467 |  |
|  | Personal Wellbeing Index (PWI) | SF-6D |  | Various | 1177-1467 |  |
|  | Satisfaction with Life Survey (SWLS) | SF-6D |  | Various | 1177-1467 |  |
|  | EQ-5D | 15D |  | Various | 1177-1467 |  |
|  | HUI3 | 15D |  | Various | 1177-1467 |  |
|  | SF-6D | 15D |  | Various | 1177-1467 |  |
|  | QWB | 15D |  | Various | 1177-1467 |  |
|  | AQoL-4D | 15D |  | Various | 1177-1467 |  |
|  | AQoL-8D | 15D |  | Various | 1177-1467 |  |
|  | Personal Wellbeing Index (PWI) | 15D |  | Various | 1177-1467 |  |
|  | Satisfaction with Life Survey (SWLS) | 15D |  | Various | 1177-1467 |  |
|  | EQ-5D | QWB |  | Various | 1177-1467 |  |
|  | HUI3 | QWB |  | Various | 1177-1467 |  |
|  | SF-6D | QWB |  | Various | 1177-1467 |  |
|  | 15D | QWB |  | Various | 1177-1467 |  |
|  | AQoL-4D | QWB |  | Various | 1177-1467 |  |
|  | AQoL-8D | QWB |  | Various | 1177-1467 |  |
|  | Personal Wellbeing Index (PWI) | QWB |  | Various | 1177-1467 |  |
|  | Satisfaction with Life Survey (SWLS) | QWB |  | Various | 1177-1467 |  |
|  | EQ-5D | AQoL-4D |  | Various | 1177-1467 |  |
|  | HUI3 | AQoL-4D |  | Various | 1177-1467 |  |
|  | SF-6D | AQoL-4D |  | Various | 1177-1467 |  |
|  | 15D | AQoL-4D |  | Various | 1177-1467 |  |
|  | QWB | AQoL-4D |  | Various | 1177-1467 |  |
|  | AQoL-8D | AQoL-4D |  | Various | 1177-1467 |  |
|  | Personal Wellbeing Index (PWI) | AQoL-4D |  | Various | 1177-1467 |  |
|  | Satisfaction with Life Survey (SWLS) | AQoL-4D |  | Various | 1177-1467 |  |
|  | EQ-5D | AQoL-8D |  | Various | 1177-1467 |  |
|  | HUI3 | AQoL-8D |  | Various | 1177-1467 |  |
|  | SF-6D | AQoL-8D |  | Various | 1177-1467 |  |
|  | 15D | AQoL-8D |  | Various | 1177-1467 |  |
|  | QWB | AQoL-8D |  | Various | 1177-1467 |  |
|  | AQoL-4D | AQoL-8D |  | Various | 1177-1467 |  |
|  | Personal Wellbeing Index (PWI) | AQoL-8D |  | Various | 1177-1467 |  |
|  | Satisfaction with Life Survey (SWLS) | AQoL-8D |  | Various | 1177-1467 |  |
| Rive, B., Grishchenko, M., Guilhaume-Goulant, C., Katona, C., Livingston, G., Lamure, M., Toumi, M., & Francois, C. (2010). Cost effectiveness of memantine in Alzheimer's disease in the UK. J Med Econ, 13(2), 371-380. | Alzheimer's Disease Cooperative Study Activities of Daily Living scale (ADCS–ADL) | EQ-5D | Alzheimer's disease | Mental health and behavioural disorders | <117 | GLM |
| Rivero-Arias O., Ouellet M., Gray A., Wolstenholme J., Rothwell P. M., Luengo-Fernandez R. (2010). Mapping the modified Rankin scale (mRS) measurement into the generic EuroQol (EQ-5D) health outcome. Med Decis Making. 30 (3), 341-54. | Modified Rankin Scale (mRS) | EQ-5D | Stroke and TIA | Cardiovascular | 2,425 | OLS; response mapping |
| Rivero-Arias, O., Ramos-Goni, J., Hernandez, M., & Gray, A. (2013). Response mapping method between SF-12 responses to EQ-5D utility values: a precisely specified algorithm and comparisons of methods to predict utilities. Presented at Warwick Medical School, University of Warwick, September 2012 and at the Centre for Research in Health and Economics (CRES), October 2012, Barcelona. Available from the authors on request. | SF-12 | EQ-5D | General population | General population | 19,678 | OLS; 2-part; CLAD; response mapping; limited dependent variable mixture model |
| Rowen D., Brazier J., Roberts J. (2009). Mapping SF-36 onto the EQ-5D index: how reliable is the relationship? Health Qual Life Outcomes. 7, 27. | SF-36 | EQ-5D | Hospital inpatients and outpatients with any condition | Various | 33248 | CLAD; Tobit; random effects GLS |
| Rowen D., Brazier J., Tsuchiya A., Alava M. H. (2012). Valuing states from multiple measures on the same visual analogue sale: a feasibility study. Health Econ. 21 (6), 715. | SF-6D | EQ-5D | General population | General population | 12048 | OLS; Utilities were mapped via VAS valuations of multiple health states, not patients' valuations |
|  | HUI2 | EQ-5D | General population | General population | 12048 |  |
|  | AQL-5D (asthma‐specific preference-based measure based on Asthma Quality of Life Questionnaire) | EQ-5D | General population | General population | 12048 |  |
|  | OPUS | EQ-5D | General population | General population | 12048 |  |
|  | ICECAP | EQ-5D | General population | General population | 12048 |  |
|  | EQ-5D | SF-6D | General population | General population | 12048 |  |
|  | HUI2 | SF-6D | General population | General population | 12048 |  |
|  | AQL-5D (asthma‐specific preference-based measure based on Asthma Quality of Life Questionnaire) | SF-6D | General population | General population | 12048 |  |
|  | OPUS | SF-6D | General population | General population | 12048 |  |
|  | ICECAP | SF-6D | General population | General population | 12048 |  |
|  | EQ-5D | HUI2 | General population | General population | 12048 |  |
|  | AQL-5D (asthma‐specific preference-based measure based on Asthma Quality of Life Questionnaire) | HUI2 | General population | General population | 12048 |  |
|  | OPUS | HUI2 | General population | General population | 12048 |  |
|  | ICECAP | HUI2 | General population | General population | 12048 |  |
|  | SF-6D | HUI2 | General population | General population | 12048 |  |
| Sauerland S., Weiner S., Dolezalova K., Angrisani L., Noguera C. M., Garcia-Caballero M., et al. (2009). Mapping utility scores from a disease-specific quality-of-life measure in bariatric surgery patients. Value Health. 12 (2), 364-70. | Moorehead-Ardelt II questionnaire (MA-II) | EQ-5D | After bariatric surgery for morbid obesity | Digestive system | 414 | OLS |
|  | Moorehead-Ardelt II questionnaire (MA-II) | SF-6D | After bariatric surgery for morbid obesity | Digestive system | 368 | OLS |
| Serrano-Aguilar P., Ramallo-Farina Y., Trujillo-Martin Mdel M., Munoz-Navarro S. R., Perestelo-Perez L., de las Cuevas-Castresana C. (2009). The relationship among mental health status (GHQ-12), health related quality of life (EQ-5D) and health-state utilities in a general population. Epidemiol Psichiatr Soc. 18 (3), 229-39. | General Health Questionnaire (GHQ-12) | EQ-5D | General population | General population | 3,032 | OLS |
| Siani, C., De Peretti, C., Millier, A., Boyer, L., & Toumi, M. (2013). Predictive models for utility from positive and negative syndrome scale clinical questionnaires for schizophrenia in the United Kingdom, France and Germany – findings of the European schizophrenia cohort (EuroSC). Discussion paper from the Laboratoire de Sciences Actuarielle et Financiere Retrieved 19th July 2013, from http://docs.isfa.fr/labo/2013.10.pdf | Positive and negative syndrome scale (PANSS) | EQ-5D | Schizophrenia | Mental health and behavioural disorders | 1208 patients | response mapping; random effects |
|  | Positive and negative syndrome scale (PANSS), Calgary Depression Scale for Schizophrenia (CDSS), Global Assessment of Functioning (GAF) and Barnes Akathisia Scale (BAS) | EQ-5D | Schizophrenia | Mental health and behavioural disorders | 1208 patients | random effects |
| Siani C., de Peretti C., Castelli C., Duru G., Daures J.-P. (2012). Uncertainty around the Incremental Cost Utility Ratio Accounting for Mapping Prediction: Application to Hepatitis C. Presented at the third joint meeting of the Health Economics Study Group and College des Économistes de la Santé meeting, January 2012, Aix-en-Provence, France. http://www.ces-asso.org/sites/default/files/ArtCS2.pdf (Accessed: 7th January 2013). | Nottingham Health Profiles (NHP) | EQ-5D | Hepatitis C | Infectious disease | 96 patients | OLS |
| Sidovar, M. F., Limone, B. L., Lee, S., & Coleman, C. I. (2013). Mapping the 12-item multiple sclerosis walking scale to the EuroQol 5-dimension index measure in North American multiple sclerosis patients. BMJ Open, 3(5), e002798. | 12-item Multiple Sclerosis Walking Scale (MSWS-12) | EQ-5D | Multiple sclerosis | Central nervous system | 1,752 | OLS |
| Soini E. J., Hallinen T. A., Puolakka K., Vihervaara V., Kauppi M. J. (2012). Cost-effectiveness of adalimumab, etanercept, and tocilizumab as first-line treatments for moderate-to-severe rheumatoid arthritis. J Med Econ. 15 (2), 340-51 and Ducournau, P., Kielhorn, A., & Wintfeld, N. (2009). Comparison of linear and nonlinear utility mapping between HAQ and EQ-5D using pooled data from the tocilizumab trials OPTION and LITHE. Rheumatology (Oxford), 48(1 Suppl), i107-108. | Health assessment questionnaire (HAQ) | EQ-5D | Rheumatoid arthritis | Musculoskeletal | 1,812 | Mixed model |
| Stahl E., Lindberg A., Jansson S. A., Ronmark E., Svensson K., Andersson F., et al. (2005). Health-related quality of life is related to COPD disease severity. Health Qual Life Outcomes. 3, 56. AND Oba Y. (2007). Cost-effectiveness of long-acting bronchodilators for chronic obstructive pulmonary disease. Mayo Clin Proc. 82 (5), 575-82. | St. George's Respiratory Questionnaire (SGRQ) | EQ-5D | Chronic obstructive pulmonary disease | Respiratory system | 168 | OLS |
| Starkie H. J., Briggs A. H., Chambers M. G., Jones P. (2011). Predicting EQ-5D values using the SGRQ. Value Health. 14 (2), 354-60. | St. George's Respiratory Questionnaire (SGRQ) | EQ-5D | Chronic obstructive pulmonary disease | Respiratory system | 14,612 | OLS; GLM; 2-part |
| Sullivan P. W., Ghushchyan V. (2006). Mapping the EQ-5D index from the SF-12: US general population preferences in a nationally representative sample. Med Decis Making. 26 (4), 401-9. | SF-12 | EQ-5D | General population | General population | 23,647 | OLS; CLAD; Tobit |
| Tsuchiya A., Brazier J., McColl E., Parkin D. (2001). A condition-specific instrument, a generic instrument, and a preference based generic instrument. Presented at the 17th Plenary Meeting of the EuroQoL Group in Copenhagen, Denmark in September 2001. http://www.euroqol.org/uploads/media/Proc01Copen2Tsuchiya.pdf (Accessed: 20th December 2012). AND Tsuchiya A., Brazier J., McColl E., Parkin D. (2002). Deriving preference-based single indices from non-preference based condition-specific instruments: Converting AQLQ into EQ5D indices. Sheffield Health Economics Group Discussion Paper Series 02/1. http://www.shef.ac.uk/content/1/c6/01/63/29/02_1FT.pdf (Accessed: 11th April 2011). | Asthma Quality of Life Questionnaire (AQLQ) | EQ-5D | Asthma | Respiratory system | 6,939 | OLS; GLM; response mapping |
| van Exel N. J., Scholte op Reimer W. J., Koopmanschap M. A. (2004). Assessment of post-stroke quality of life in cost-effectiveness studies: the usefulness of the Barthel Index and the EuroQoL-5D. Qual Life Res. 13 (2), 427-33. | Barthel index | EQ-5D | Stroke | Cardiovascular | 710 | OLS |
| van Hout B., Janssen M. F., Feng Y. S., Kohlmann T., Busschbach J., Golicki D., et al. (2012). Interim scoring for the EQ-5D-5L: mapping the EQ-5D-5L to EQ-5D-3L value sets. Value Health. 15 (5), 708-15. | EQ-5D-5L | EQ-5D | "Broad spectrum of health" including people with stroke, rheumatoid arthritis and personality disorder | Various | 3,691 | OLS; response mapping; non-parametric cross-tabulation; psychometric scaling approach |
| Versteegh M. M., Leunis A., Luime J. J., Boggild M., Uyl-de Groot C. A., Stolk E. A. (2012). Mapping QLQ-C30, HAQ, and MSIS-29 on EQ-5D. Med Decis Making. 32 (4), 554-68. | EORTC Quality of Life Questionnaire (QLQ-C30) | EQ-5D | Multiple myeloma and non-Hodgkin lymphoma | Cancer | 661 | OLS |
|  | Health assessment questionnaire (HAQ) | EQ-5D | Arthritis | Musculoskeletal | 186 | OLS |
|  | Health Assessment Questionnaire (HAQ), SF-36, Hospital Anxiety and Depression Scale (HADS) & Disease Activity Score (DAS28) | EQ-5D | Arthritis | Musculoskeletal | 186 | OLS |
|  | MSIS-29 | EQ-5D | Multiple sclerosis | Central nervous system | 723 | OLS |
| Versteegh M. M., Rowen D., Brazier J. E., Stolk E. A. (2010). Mapping onto Eq-5 D for patients in poor health. Health Qual Life Outcomes. 8, 141. | Health Assessment Questionnaire (HAQ) | EQ-5D | Patients with and without rheumatoid arthritis | Various | 493 | OLS |
| Whynes, D. K., Sprigg, N., Selby, J., Berge, E., & Bath, P. M. (2013). Testing for Differential Item Functioning within the EQ-5D. Med Decis Making, 33(2), 252-260. | Modified Rankin Scale (mRS) | EQ-5D | Stroke | Cardiovascular | 1462 patients | OLS |
|  | Modified Rankin Scale (mRS), Barthel Index and Zung Depression | EQ-5D | Stroke | Cardiovascular | 1462 patients | OLS |
| Wijeysundera H. C., Tomlinson G., Norris C. M., Ghali W. A., Ko D. T., Krahn M. D. (2011). Predicting EQ-5D utility scores from the Seattle Angina Questionnaire in coronary artery disease: a mapping algorithm using a Bayesian framework. Med Decis Making. 31 (3), 481-93. | Seattle Angina Questionnaire (SAQ) | EQ-5D | Coronary artery disease | Cardiovascular | 1,555 | OLS; Tobit |
| Williamson I., Benge S., Barton S., Petrou S., Letley L., Fasey N., et al. (2009). A double-blind randomised placebo-controlled trial of topical intranasal corticosteroids in 4- to 11-year-old children with persistent bilateral otitis media with effusion in primary care. Health Technol Assess. 13 (37), 1-144. AND Dakin H., Petrou S., Haggard M., Benge S., Williamson I. (2010). Mapping analyses to estimate health utilities based on responses to the OM8-30 Otitis Media Questionnaire. Qual Life Res. 19 (1), 65-80. | OM8-30 (disease-specific otitis media instrument) | EQ-5D | Children with otitis media | Ear, nose and throat | 212 | OLS |
|  |  | HUI2 | Children with otitis media | Ear, nose and throat | 218 | OLS; GLM; 2-part |
|  |  | HUI3 | Children with otitis media | Ear, nose and throat | 205 | OLS; GLM; 2-part |
| Wolfe F., Michaud K., Wallenstein G. (2010). Scale characteristics and mapping accuracy of the US EQ-5D, UK EQ-5D, and SF-6D in patients with rheumatoid arthritis. J Rheumatol. 37 (8), 1615-25. | Health Assessment Questionnaire (HAQ) | EQ-5D | Rheumatoid arthritis | Musculoskeletal | 10,895 | OLS; fractional polynomial regression |
|  |  | SF-6D | Rheumatoid arthritis | Musculoskeletal | 10,895 | OLS; fractional polynomial regression |
| Wu E. Q., Mulani P., Farrell M. H., Sleep D. (2007). Mapping FACT-P and EORTC QLQ-C30 to patient health status measured by EQ-5D in metastatic hormone-refractory prostate cancer patients. Value Health. 10 (5), 408-14. | Functional Assessment of Cancer Therapy-Prostate (FACT-P) | EQ-5D | Prostate cancer | Cancer | 276 | OLS; 2-part; median regression |
|  | FACT-P and EORTC Quality of Life Questionnaire (QLQ-C30) | EQ-5D | Prostate cancer | Cancer | 276 | OLS; 2-part; median regression |
| Xie F., Pullenayegum E. M., Li S. C., Hopkins R., Thumboo J., Lo N. N. (2010). Use of a disease-specific instrument in economic evaluations: mapping WOMAC onto the EQ-5D utility index. Value Health. 13 (8), 873-8. | Western Ontario and McMaster Universities Osteoarthritis Index (WOMAC) | EQ-5D | Knee osteoarthritis | Musculoskeletal | 258 | OLS; CLAD |
| Young, M. K., Ng, S. K., Mellick, G., & Scuffham, P. A. (2013). Mapping of the PDQ-39 to EQ-5D scores in patients with Parkinson's disease. Qual Life Res, 22(5), 1065-1072. | 39-item Parkinson’s Disease Questionnaire (PDQ-39) | EQ-5D | Parkinson's disease | Central nervous system | 80 | OLS; response mapping |
